# The structural and chemical basis of temporary adhesion in the sea star *Asterina gibbosa*

**DOI:** 10.3762/bjnano.9.196

**Published:** 2018-07-30

**Authors:** Birgit Lengerer, Marie Bonneel, Mathilde Lefevre, Elise Hennebert, Philippe Leclère, Emmanuel Gosselin, Peter Ladurner, Patrick Flammang

**Affiliations:** 1 Biology of Marine Organisms and Biomimetics Unit, Research Institute for Biosciences, University of Mons, 23 Place du Parc, 7000 Mons, Belgium; 2 Cell Biology Unit, Research Institute for Biosciences, University of Mons, 23 Place du Parc, 7000 Mons, Belgium; 3 Laboratory for Chemistry of Novel Materials, Center for Innovation and Research in Materials and Polymers (CIRMAP), University of Mons, 20 Place du Parc, 7000 Mons, Belgium; 4 Laboratory of Physics of Surfaces and Interfaces (LPSI), University of Mons, 23 Place du Parc, 7000 Mons, Belgium; 5 Institute of Zoology and Center of Molecular Bioscience Innsbruck, University of Innsbruck, Technikerstr. 25, A-6020 Innsbruck, Austria

**Keywords:** duo-gland adhesive system, lectins, marine temporary adhesion, starfish

## Abstract

**Background:** Marine biological adhesives are a promising source of inspiration for biomedical and industrial applications. Nevertheless, natural adhesives and especially temporary adhesion systems are mostly unexplored. Sea stars are able to repeatedly attach and detach their hydraulic tube feet. This ability is based on a duo-gland system and, upon detachment, the adhesive material stays behind on the substrate as a 'footprint'. In recent years, characterization of sea star temporary adhesion has been focussed on the forcipulatid species *Asterias rubens*.

**Results:** We investigated the temporary adhesion system in the distantly related valvatid species *Asterina gibbosa*. The morphology of tube feet was described using histological sections, transmission-, and scanning electron microscopy. Ultrastructural investigations revealed two adhesive gland cell types that both form electron-dense secretory granules with a more lucid outer rim and one de-adhesive gland cell type with homogenous granules. The footprints comprised a meshwork on top of a thin layer. This topography was consistently observed using various methods like scanning electron microscopy, 3D confocal interference microscopy, atomic force microscopy, and light microscopy with crystal violet staining. Additionally, we tested 24 commercially available lectins and two antibodies for their ability to label the adhesive epidermis and footprints. Out of 15 lectins labelling structures in the area of the duo-gland adhesive system, only one also labelled footprints indicating the presence of glycoconjugates with α-linked mannose in the secreted material.

**Conclusion:** Despite the distant relationship between the two sea star species, the morphology of tube feet and topography of footprints in *A. gibbosa* shared many features with the previously described findings in *A. rubens*. These similarities might be due to the adaptation to a benthic life on rocky intertidal areas. Lectin- and immuno-labelling indicated similarities but also some differences in adhesive composition between the two species. Further research on the temporary adhesive of *A. gibbosa* will allow the identification of conserved motifs in sea star adhesion and might facilitate the development of biomimetic, reversible glues.

## Introduction

Marine biological adhesives are environmentally friendly, biodegradable, and adhere to various surfaces in the challenging conditions of the sea [1]. These features make them ideal templates for biomimetic glues. However, only few marine adhesives have been characterized so far (reviewed in [2–4]). The best-investigated glues are produced by sessile organisms like mussels, tubeworms, and barnacles (reviewed in [5–7]). Although single proteins of marine temporary adhesives have been identified [8–10], non-permanent adhesion remains poorly understood. Echinoderms represent promising organisms to study reversible adhesion, their hydraulic tube feet being able to repeatedly attach and detach [11–12]. All echinoderm tube feet consist of four tissue layers: an inner myomesothelium, a connective tissue layer, a nerve plexus, and an outer epidermis. The shape of tube feet is highly variable, but in terms of adhesion the disc-ending tube feet are of particular interest [11,13–14]. These tube feet consist of a flexible stem and an enlarged, flattened disc [11]. At the level of the disc, the epidermis is specialized into a sensory-secretory epithelium, enabling perception and adhesion [11–12]. For several echinoderm species, the adhesive strength was estimated by measuring the tenacity of single tube feet [13,15–18]. The measured tenacity ranged from 0.2 MPa in the sea star *Asterias rubens* [15], up to 0.54 MPa in the sea urchin *Colobocentrotus atratus* [19], indicating a strong attachment to the substrate. Moreover, when well-attached sea stars and sea urchins are forcefully pulled from the substrate, many of their tube feet break, leaving their discs and part of the stems attached [17,19–20]. These observations proved that the tenacity of the produced glue can exceed the tensile strength of the stem.

The strong and temporary adhesion of echinoderms was proposed to rely on a duo-gland adhesive system [21–23]. In duo-gland adhesive systems the adhesive gland cells secrete the glue and a different gland cell type produces a de-adhesive substance. Additional supportive cells enclosing a prominent bundle of intermediate filaments provide the required mechanical strength [24]. Upon voluntary detachment, the adhesive substance is left behind on the substrate as a 'footprint' of approximately the same diameter as the tube feet [11–12,25–26]. In echinoderm footprints, the organic part consists of mainly proteins and carbohydrates [22,27]. The footprints are built by the overlay of a thin homogeneous film covering the substrate with a sponge-like meshwork on top [11–12,25–26]. This topography is not altered by the release of the de-adhesive substance [26].

In the forcipulatid sea star *Asterias rubens,* adhesive secretions were investigated in greater detail. In this species, the footprint material is produced by two adhesive gland cell types [25]. The content of type 2 adhesive cells is secreted first, and is supposed to form the thin homogeneous film. The content of type 1 adhesive cells forms the thick meshwork and provides the cohesive strength [26]. One protein present in the meshwork was recently characterized and named sea star footprint protein-1 (Sfp1) [8]. Thirty-four additional proteins specific for footprints were identified and at least two were found to be glycosylated [28–29]. Additionally, lectin labelling of tube foot sections and footprints in *A. rubens* revealed the presence of various sugar moieties (*N*-acetylgalactosamine, *N*-acetylglucosamine, galactose, mannose and glucose residues) within the adhesive material [28]. Among sea stars, the protein and carbohydrate composition of the adhesive material has been solely investigated in the species *A. rubens*. Yet, polyclonal antibodies raised against footprint material of *A. rubens* led to a strong immunolabelling within the adhesive epidermis of thirteen other asteroid species [14].

The characterization of adhesive tube feet and footprint material in different asteroid species will help to identify shared features of temporary adhesives in sea stars and might increase our understanding thereof. In this paper, we investigated the structural and chemical basis of temporary adhesion in the valvatid species *Asterina gibbosa*. The most recent molecular phylogeny of the Class Asteroidea supports a tree in which two main groups apparently diverged early in the evolution of sea stars [30]. According to this phylogeny, *A. gibbosa* and *A. rubens* could be considered as distantly-related species as they each belong to one of these two main sea star clades. We characterized the morphology of *A. gibbosa* tube feet using light microscopy, and transmission- and scanning electron microscopy (TEM, SEM). The cell types and intracellular structures of the adhesive epidermis were described and compared to other sea star species. The topography of *A. gibbosa* footprints deposited on glass slides was investigated with SEM, 3D confocal interference microscopy, and atomic force microscopy (AFM). *A. gibbosa* tube feet and footprints were labelled with antibodies raised against the adhesive protein Sfp1 from *A. rubens,* but no cross-reactivity was observed. To detect carbohydrate moieties, we performed lectin labelling with 24 commercially available lectins on tube foot sections and footprints.

## Results and Discussion

**Figure 1:**
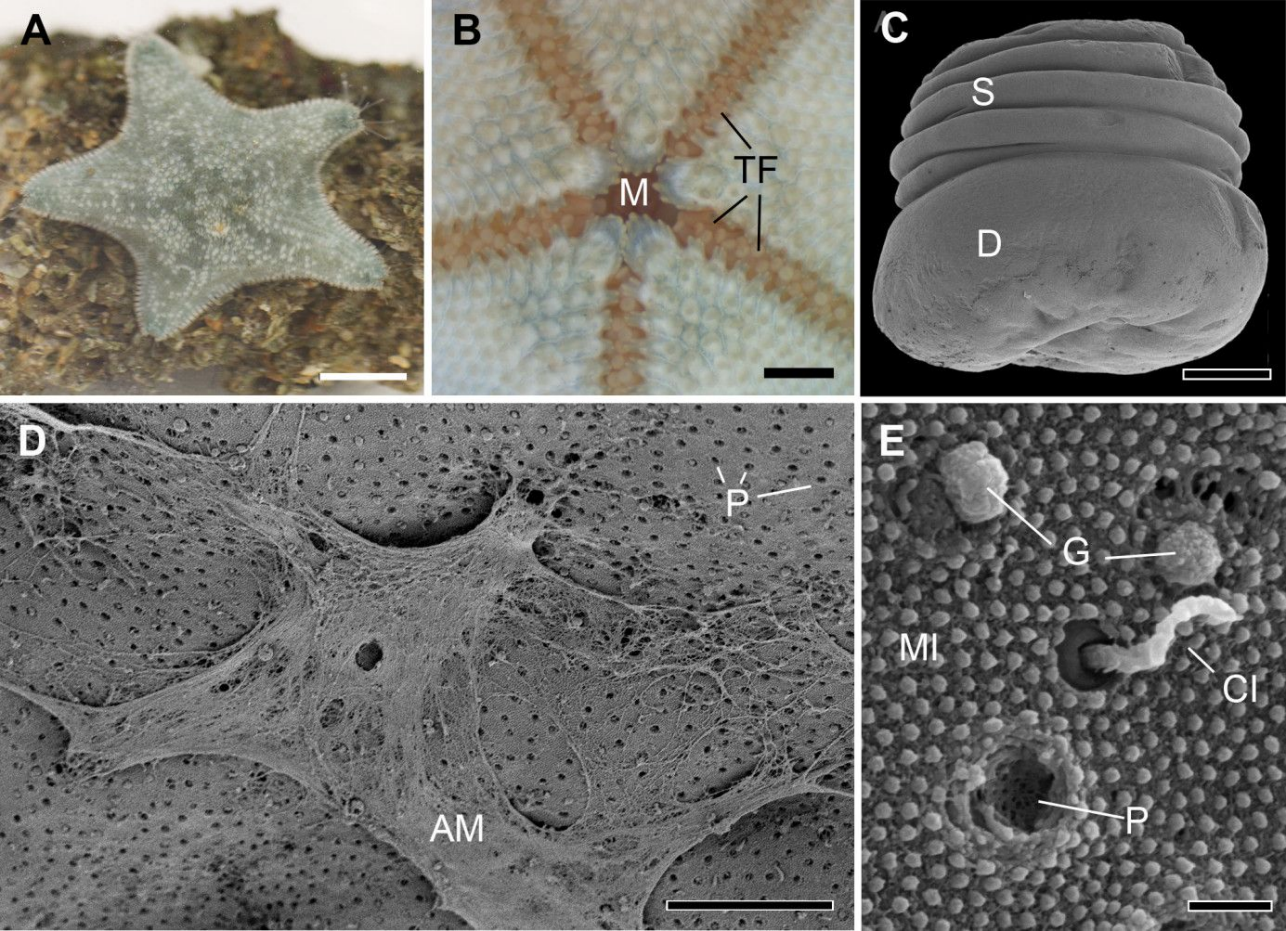
External morphology of the sea star *Asterina gibbosa* and of its tube feet. (A) Image of a living adult, attached to a rock. (B) Oral side of an adult, showing the arrangement of the tube feet in the ambulacral grooves along the five arms. (C) Overview of an amputated tube foot with SEM. (D) SEM image of the disc distal surface with a layer of adhesive material. (E) Details of secretory pores and cilia (SEM). AM - adhesive material; CI - cilia; D - disc; G - granule; M - mouth; MI - microvilli; P - pores; S - stem; TF - tube feet. Scale bars: (A) 1 cm; (B) 0.2 cm; (C) 100 µm; (D) 10 µm; (E) 0.5 µm.

The starlet cushion star, *Asterina gibbosa*, is a small sea star inhabiting wide areas in the northeast Atlantic Ocean and the Mediterranean Sea (Figure 1A). Being an exclusively benthic animal, it relies on the strong and reversible attachment achieved through the hundreds of tube feet arranged in double rows on the oral surface of each arm (Figure 1B).

### Morphology and ultrastructure of the tube feet

**External morphology.** The folded stem and the flattened adhesive disc were clearly distinguishable in SEM images (Figure 1C). For SEM preparation, individual tube feet were amputated and only a part of the stem was maintained (Figure 1C). On some tube feet, adhesive material was preserved on the disc surface (Figure 1D). The material appeared fibrous and emerged from secretory pores. Fibrils originated from single pores and clustered together to form a layer of adhesive material (Figure 1D). Clean disc surfaces showed the evenly distributed secretory pores and sensory cilia (Figure 1E). Both structures were present throughout the whole distal area of the tube foot disc. Often adhesive granules were observed emerging from the secretory pores (Figure 1E). Short microvilli completely covered the disc surface (Figure 1E).

**Figure 2:**
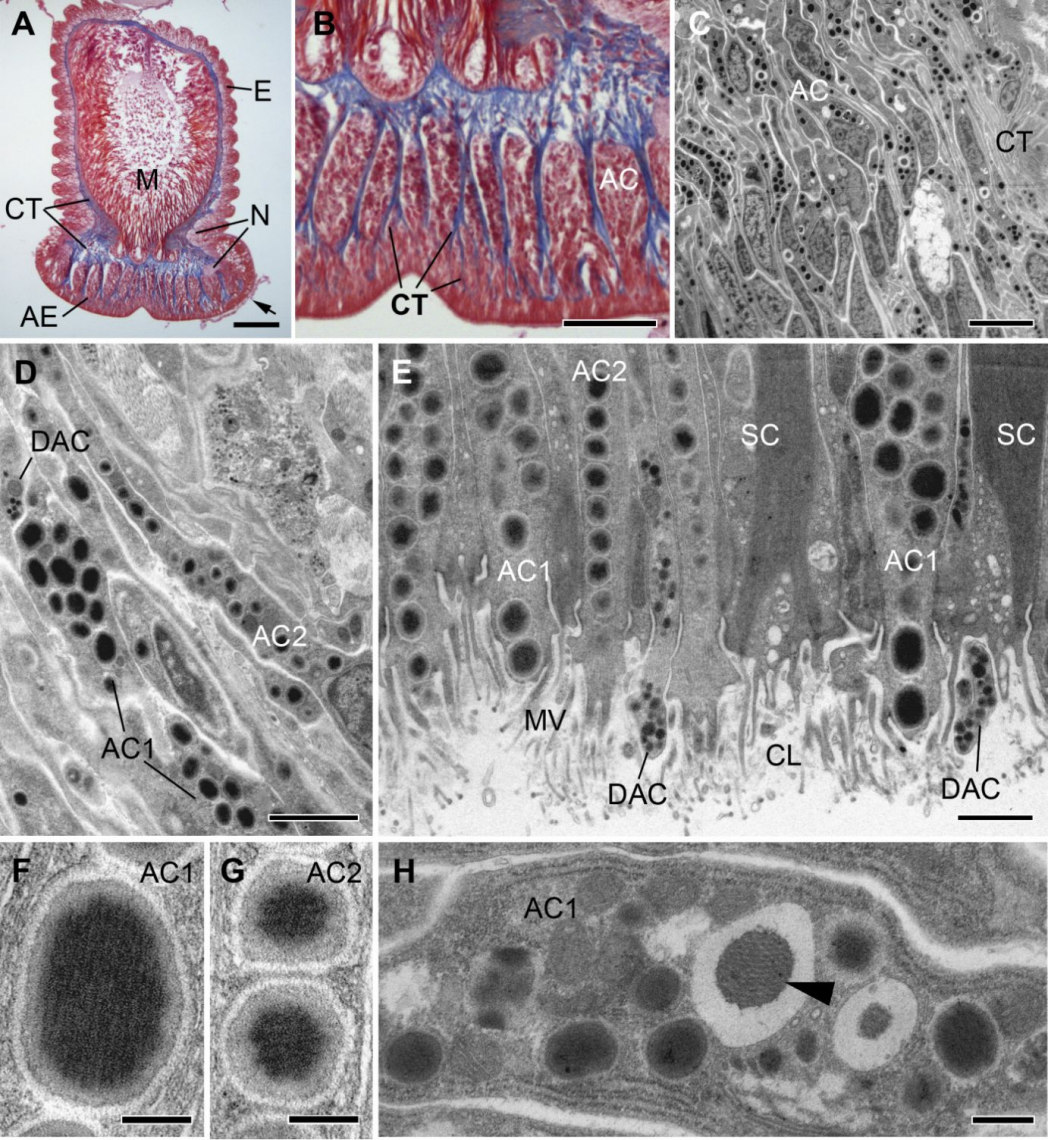
Fine structure of the tube feet of *Asterina gibbosa* observed in light microscopy (A,B) and TEM (C–H). (A,B) Longitudinal histological section through a tube foot stained with Heidenhain’s azan trichrome. Arrow highlights remains of adhesive material. (C–H) TEM images of longitudinal tube foot sections, arrowhead in (H) indicates inner core of secretory granule. See text for details. AC - adhesive gland cell; AE - adhesive epidermis; CL - cuticular layer; CT - connective tissue; DAC - de-adhesive gland cell; E - epidermis; M - myomesothelium; MV - microvilli; N - nerve strands; SC - supportive cell. Scale bars: (A) 100 µm; (B) 50 µm; (C) 5 µm; (D) 2 µm; (E) 1 µm; (F,G) 0.2 µm; (H) 0.5 µm.

**Figure 3:**
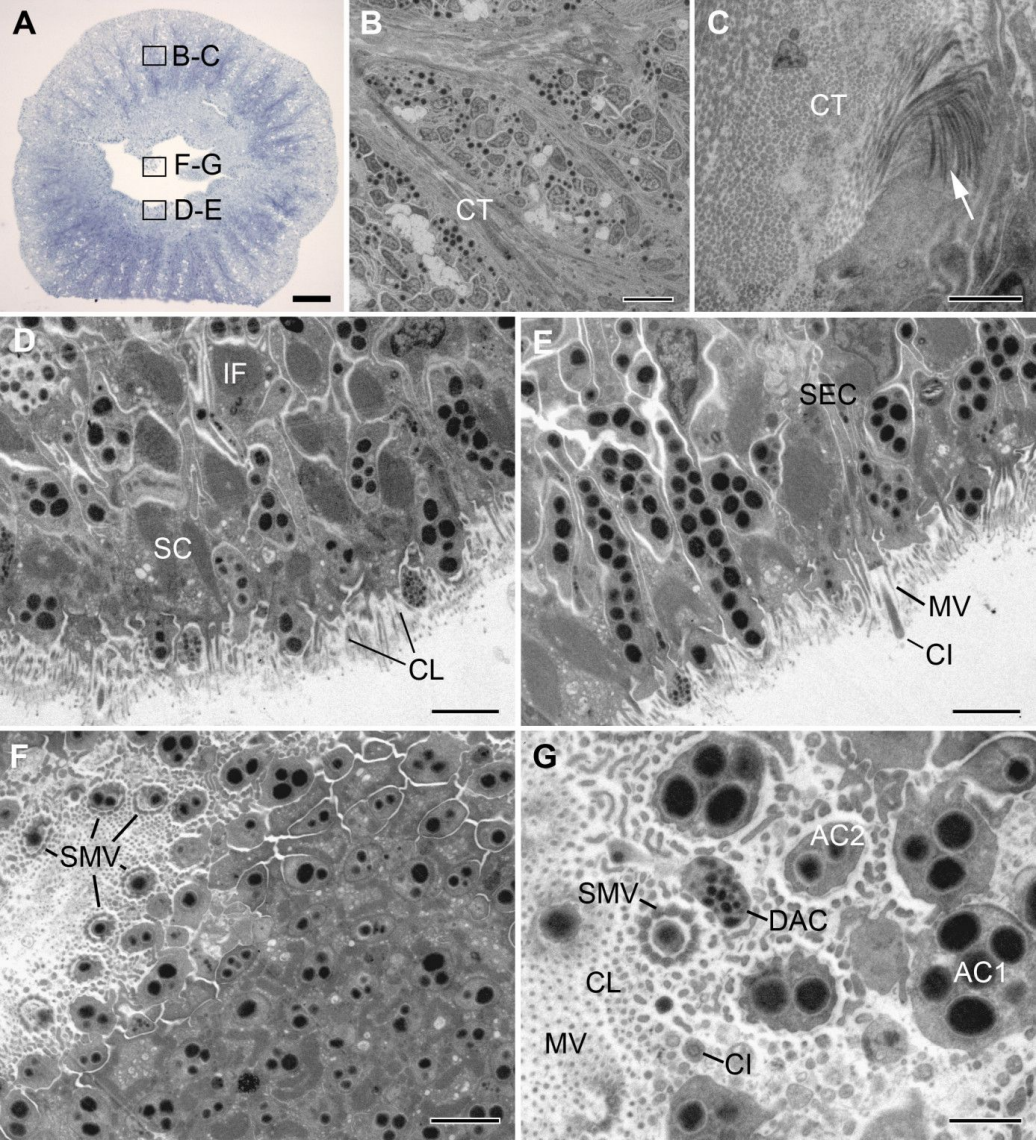
Ultrastructure of the tube foot adhesive epidermis of *Asterina gibbosa* observed in light microscopy (A) and TEM (B–G). (A) Semi thin cross-section of a tube foot at the level of the disc, boxes indicate approximate area of TEM images. (B–G) Ultrastructure of cells in the adhesive epidermis: (B,C) in the basal part of the disc, at the level of the connective tissue, and (D–G) in the apical part of the disc. Arrow in (C) highlights the collagen fibres of the connective tissue. AC - adhesive gland cell; CI - cilia; CL - cuticular layer; CT - connective tissue; DAC - de-adhesive gland cell; IF - intermediate filaments; MV - microvilli; SC - supportive cell; SEC - sensory cell; SMV - specialized microvilli. Scale bars: (A) 50 µm; (B) 5 µm; (C,G) 1 µm; (D,E,F) 2 µm.

**Histology of the inner tissues.** On histological sections, the four tube foot specific tissue layers were observed and consisted of an inner myomesothelium that encircled the inner lumen, a connective tissue layer, nerve strands, and an outer epidermis covered by a thin glycocalyx, the so-called cuticle (Figure 2A). On some histological sections, the adhesive material, also visible on SEM pictures, was preserved on the adhesive epidermis (Figure 2A). As characteristic for reinforced disc-ending tube feet, the disc of *A. gibbosa* was slightly broader than the flexible stem and full of collagenous fibres (Figure 2B). The collagen fibres were arranged in thick bundles and alternated with clusters of secretory gland cells (Figure 2B and Figure 3A). In asteroids, three tube foot morphotypes have been described – i.e., knob-ending tube feet, disc-ending tube feet, and reinforced disc-ending tube feet – and it has been predicted that the morphology of tube feet is more influenced by adaptations to the habitat than by evolutionary lineage [14,31]. Adults of *A. gibbosa* can be found in crevices or under boulders on rocky shores. The distantly-related forcipulatid species *A. rubens* inhabits a wide range of habitats and is also commonly found in rocky intertidal areas. Both species possess reinforced disc-ending tube feet and their morphology appears similar in histological sections [14].

**Ultrastructure of the adhesive epidermis.** In most asteroids a duo-gland adhesive system with two adhesive gland cell types and one de-adhesive gland type is present [11]. In *A. gibbosa*, the secretory gland cell bodies of the duo-gland adhesive system were located in the basal part of the disc epidermis and sent long necks to the disk surface (Figure 2B–E). At the apical area of the disc, secretory gland cells were intermingled with supportive cells. The surface of the disc epidermis, the area of secretion, was covered with short microvilli (Figure 1E) also visible in SEM. The secretory gland cells were filled with densely-packed membrane-bound granules. Based on the granules size and appearance, three gland cell types could be distinguished (Figure 2D,E). The de-adhesive gland cells of *A. gibbosa* formed characteristic small electron dense secretory granules of approximately 125 ± 17 nm (*n* = 34) in diameter (Figure 1E) (numbers given are average diameter with standard deviations and number of measured granules). The de-adhesive granule appearance is in line with results obtained in other asteroid species [11,32]. Two other secretory cell types were also recognized and both contained granules with a very electron dense inner core and a less electron dense outer rim (Figure 2F,G). Based on observations in other sea star species [11], we classified these cells as adhesive gland cell type 1 and 2. The type 1 adhesive granules were ellipsoid with measures of 596 ± 68 nm along the major axis and 431 ± 41 nm (*n* = 38) along the minor axis (Figure 2E,F). In contrast, type 2 adhesive granules were globular and, with a diameter of 346 ± 47 nm (*n* = 30), smaller than type 1 granules (Figure 2E,G). These differences were more apparent in longitudinal sections than in cross sections of tube feet, indicating that the ellipsoid granules were oriented with their major axis along the gland necks. In type 1 adhesive granules, dense parallel oriented fibres could be distinguished (Figure 2F), whereas the inner core of type 2 granules appeared homogenous (Figure 2G). The fibrillary structures in type 1 adhesive granules were more obvious in newly forming granules (Figure 2H). These condensing secretory granules were common in the basal part of the epidermis, at the level of the cell bodies (Figure 2C). At this level, the gland cells were full of rough endoplasmic reticulum and condensing granules (Figure 2G). Along the necks and in the apical part of the epidermis only mature secretory granules were observed (Figure 2D,E and Figure 3D,E).

The tube foot discs were thinner in the centre compared to the margin (Figure 1C). Therefore, cross sections facilitated the observation of cells of the adhesive epidermis at different depths (Figure 3A). In the basal area of the disc, the connective tissue (CT) formed branched septa, enclosing bundles of secretory gland cells (Figure 3B). The connective tissue consisted mainly of collagen fibres, which are bearing the force during attachment (Figure 3C). The collagen fibres were absent in the most apical areas of the disc epidermis. There, the supportive cells take the task of providing mechanical strength (Figure 3D). At this level the supportive cells were found to be the most numerous and alternated with secretory gland necks. The cytoplasm of supportive cells was almost completely filled with densely packed intermediate filaments (Figure 3D). The adhesive gland cells clearly outnumbered the de-adhesive gland cells throughout the adhesive epidermis. In longitudinal sections, it appeared that type 1 and 2 adhesive gland cells were homogenously distributed. However, as the two types were not easily discriminated in cross sections, the relative proportion between these cells could not be determined. Between supportive cells, adhesive and de-adhesive gland necks, another non-secretory cell type, sensory cells, was observed (Figure 3E). These cells bore a single cilium, with a characteristic 9×2 + 2 microtubule structure, surrounded by a microvilli collar (Figure 3E). The morphology indicated that these cilia have sensory function and might be involved in surface exploration [12,21]. At the level of the disc surface, the three secretory cells of the duo-gland adhesive system secrete their granules through the microvilli-supported cuticular layer. In cross sections, the difference between the secretion mode of the adhesive and de-adhesive cells became obvious (Figure 3F). Both type 1 and 2 adhesive cells formed an apical secretory duct lined by specialized microvilli enforced with actin filaments (Figure 3F,G), whereas de-adhesive cells lacked this supportive structure and presented instead a simple apical granule-filled bulge (Figure 3D,E,G).

In addition to temporary adhesion with tube feet in adults, *A. gibbosa* presents other adhesion mechanisms during its life cycle. Indeed, this species has an entirely benthic and lecithotrophic development [33]. From hatching to adulthood three attachment modes can be distinguished, reversible adhesion in brachiolaria larvae, permanent attachment during metamorphosis, and finally tube feet-based temporary adhesion in adults [34]. In previous studies, the morphology and attachment strength of brachiolaria larvae, metamorphic individuals, and juveniles of *A. gibbosa* have been investigated [34–35]. Brachiolaria larvae have two arms with secretory areas at the tip. These arms are used for reversible attachment. At a later stage, the larvae form an additional adhesive disc, which they use to cement themselves to the substrate to undergo metamorphosis. The adhesive areas on the arms are covered by secretory pores with a short protruding cilium [34]. This is in contrast to the adhesive pores found in adult tube feet that lacked a cilium (Figure 1D). Furthermore, the ultrastructure of adhesive gland cells differs substantially between developmental stages. In the larval arms, only one ciliated adhesive gland cell type is present with large (approx. 1.2 µm long and 0.8 µm wide) ellipsoid granules. A low number of de-adhesive gland cells have also been described in the brachiolar arms. In the adhesive disc, an additional ciliated secretory gland cell with large, less electron-dense granules but without secretory pore has been observed. These findings indicate that the adhesive gland cells are distinct for all three attachment modes. Interestingly, larval brachiolar arms and adult tube feet, which are both involved in temporary adhesion and show similar adhesion strength [35], appear to rely on morphologically different adhesive cells but similar de-adhesive cells.

In echinoderms the appearance of tube foot adhesive secretory granules is variable and a correlation between granule ultrastructure and species habitat has been predicted in sea stars [32,36]. Echinoderm adhesive granules can be divided into five categories: (1) homogeneous granules, (2) heterogeneous granules with an irregular mixture of two materials, (3) granules with an electron-dense core and a lucid outer rim, (4) granules with an inner filamentous bundle, and (5) granules with a lucid material, which is capped on one side with an electron-dense material [11]. In *A. gibbosa,* both types of adhesive granules consisted of a dense inner core and a more electron lucid outer rim, but fibres were observed only in type 1 granules (Figure 2F,G). This classified type 1 granules as granules with an inner filamentous bundle and type 2 granules as granules with an electron-dense core and a lucid outer rim. Similar to *A. gibbosa,* type 1 adhesive granules in *A. rubens* are ellipsoid (1 µm long and 0.6 µm in diameter) and contain parallel fibres. Type 2 adhesive granules in *A. rubens* are spherical and smaller (550 nm), but in contrast to *A. gibbosa* they are less electron dense than type 1 granules [25]. This dissimilar electron-density might indicate a difference in the granule content between the two species. In *A. rubens*, the material of type 2 adhesive gland cells is secreted first, forming the contact with the substrate, and type 1 cells form the thick meshwork of the footprints [8,26]. We based our classification of type 1 and 2 adhesive granules in *A. gibbosa* on morphological features like size and shape. Currently, it is therefore unknown if the function of the two cell types is homologous to that proposed in *A. rubens*.

In many marine invertebrate species with a duo-gland adhesive system, the adhesive granules are secreted through a specialized microvilli collar [37–40]. In contrast to asteroids, the microvilli collar in flatworms is formed by supportive cells and it clearly protrudes from the surrounding epidermis. In higher flatworms, the openings are separate and similar to echinoderms only the adhesive gland necks are encircled with specialized microvilli. In basal flatworms, both adhesive and de-adhesive cells secrete through a common microvilli collar [39–40]. It was assumed that only the tip of this microvilli collar gets attached to the substrate [37]. In the basal marine flatworm *Macrostomum lignano*, impairing the morphology of the supportive cells and their microvilli collar prevented the animals from attaching themselves [37,41]. In asteroids, the area of attachment is an order of magnitude larger and completely covered by normal and specialized microvilli. The microvilli are embedded in a cuticle, which is poorly preserved in standard TEM preparations. We assume that, similar to their function in flatworms, the microvilli of sea star tube feet are involved in the attachment process and might additionally help to distribute the mechanical forces.

### Footprint structure and topography

**Figure 4:**
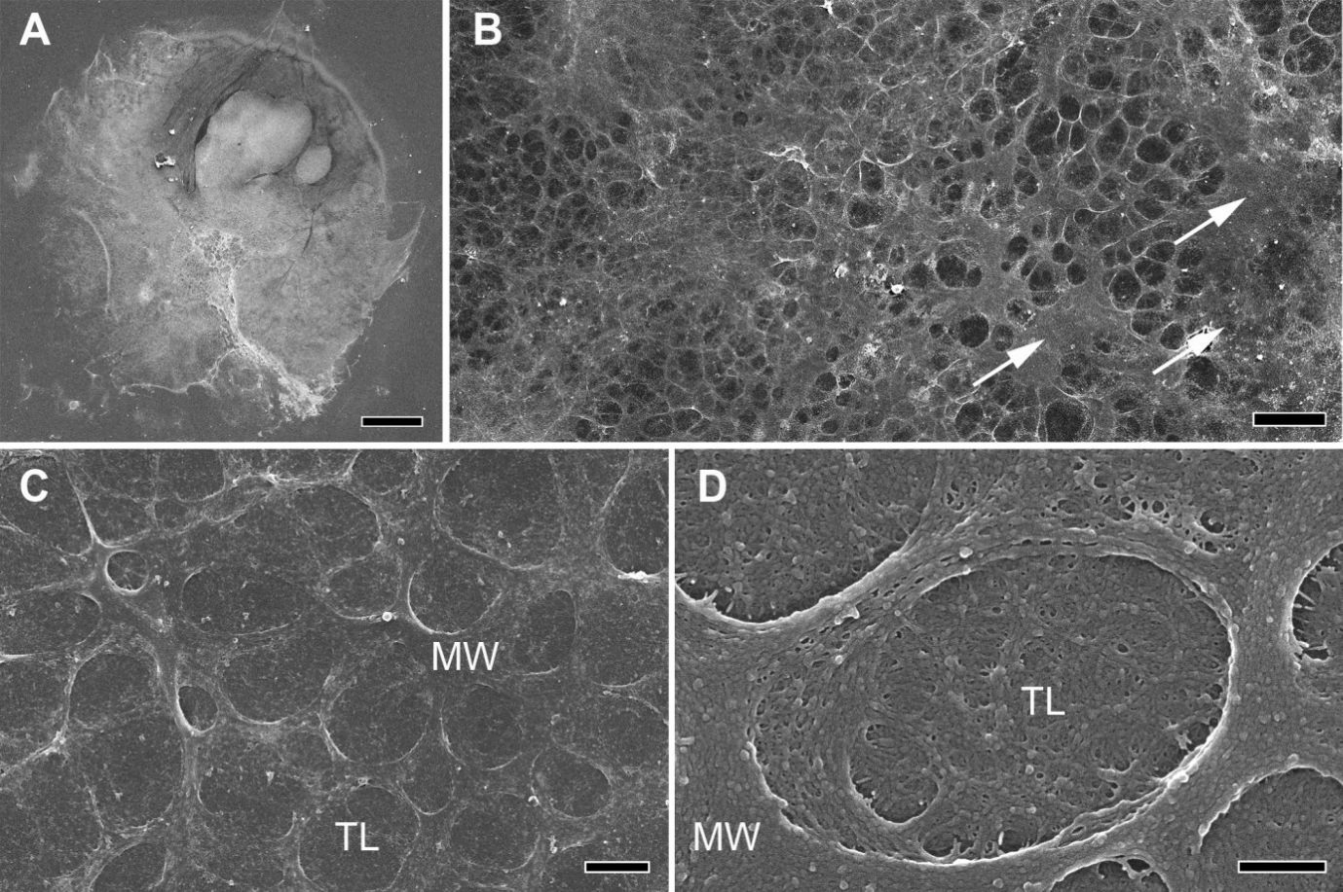
Scanning electron microscopy of footprints in *Asterina gibbosa*. (A) Overview of a complete footprint. (B) Characteristic area of a footprint with visible meshwork. Arrows indicate thicker parts, where the meshwork is not distinguishable. (C,D) Details of the meshwork at higher magnifications. MW - meshwork; TL - thin layer. Scale bars: (A) 100 µm; (B) 10 µm; (C) 2 µm; (D) 0.5 µm.

**Figure 5:**
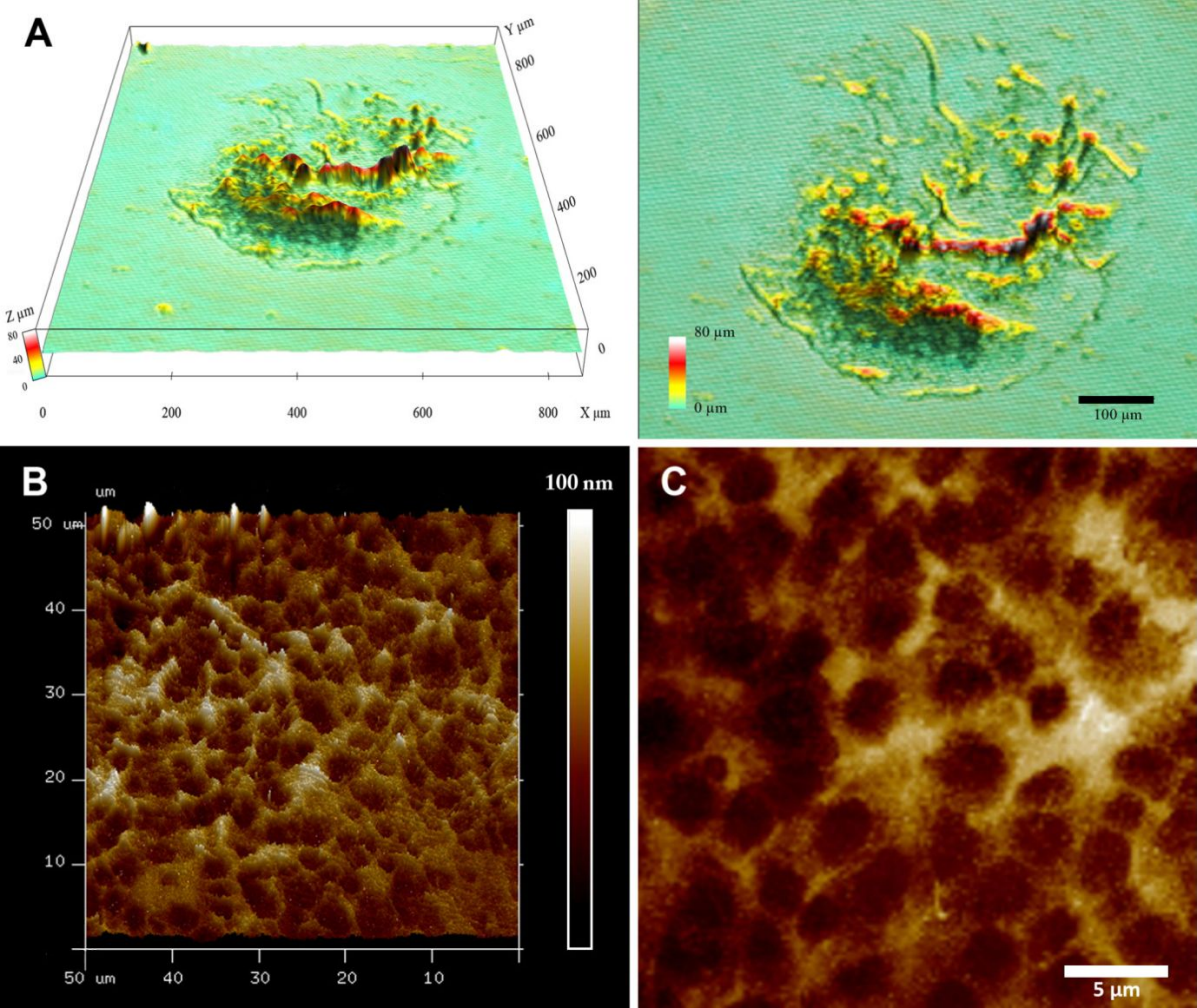
Topography of the footprints in *A. gibbosa* shown with 3D confocal interference microscopy (A) and AFM (B,C). (A) Overview of a footprint with 3D confocal interference microscopy in 3D and 2D. (B,C) AFM images of footprint meshwork in (B) 3D and at higher resolution in (C) 2D.

After voluntary detachment of the tube feet, the adhesive material was left behind on the glass slides as footprints. With SEM, the footprints appeared as roundish imprints of approximately the same diameter as the tube feet (Figure 4A). The amount of deposited material varied between individual footprints as well as within different areas of a single footprint (Figure 4A). Additionally, partial footprints were observed (not shown). In many areas of the footprints, two layers were present: a meshwork deposited on top of a thin layer of material (Figure 4B). In thicker areas, as well as in very thin ones, this meshwork was not distinguishable (Figure 4B). The mesh size varied from 1 to 5 µm in diameter (Figure 4C). At higher magnification, the fine structure of both layers appeared similar (Figure 4D). The footprint topography was confirmed with 3D confocal interference microscopy and atomic force microscopy (AFM) (Figure 5). 3D confocal interference microscopy was used to visualize the 3D structure of whole footprints. The roughness average within footprints was around 3 µm, compared to 0.9 µm outside of the footprints, where also small amounts of material were detected (Figure 5A). However, the thickness considerably varied within footprints and areas with highly stacked material occurred on the otherwise thin layer (Figure 5A). Areas with a prominent meshwork were further investigated with AFM (Figure 5B,C). The height profile through the meshwork showed that it was about 60–90 nm high (Figure 5B,C).

The topography of *A. gibbosa* footprints resembled that described in *A. rubens* [22,25–26]. Similar footprint structures have been described in many temporary adhering animals, from other sea star species [20,25], to sea urchins [12], ectoparasitic flatworms [42], and the cnidarian *Hydra* [43]. It is noteworthy that in *A. gibbosa* the described footprint structures were only observed after animals attached firmly for at least one minute to the substrate (see Experimental). When animals just walked on glass slides without agitation, no detectable footprints were observed. Therefore, we assume that adhesive strength might be adjusted by the amount of secreted material. When animals were attached strongly to glass slides, pulling them away with force (non-voluntarily detachment) commonly caused tube feet to break apart at the area of the disc or the stem. This has been observed in several other echinoderm species [17,19–20] and emphasizes the adhesive strength of the glue.

### Composition of the adhesive material

The tube foot disc of the valvatid *A. gibbosa* can be labelled by polyclonal antibodies raised against the footprint material of the forcipulatid *A. rubens* [14]. These results hint to the presence of conserved adhesive components between those two distantly-related species. In *A. rubens*, the dried footprint material consists of approximately 20.6% of proteins, 8% of carbohydrates, 5.6% of lipids, 2.5% of sulphates and 40% of inorganic residues. The composition of the remaining fraction could not be determined [22]. We used antibodies and lectins to further investigate the similarities and differences in adhesive material composition between *A. gibbosa* and *A. rubens*.

**Adhesive protein variability.** The footprints of *A. rubens* consist of a complex blend of different proteins [29] but only one of them, Sfp1, has been fully characterized to date [8]. No immunoreactivity for antibodies against Sfp1α and Sfp1β of *A. rubens* was observed in *A. gibbosa* adhesive granules and footprints (Supporting Information File 1, Figure S1).

**Figure 6:**
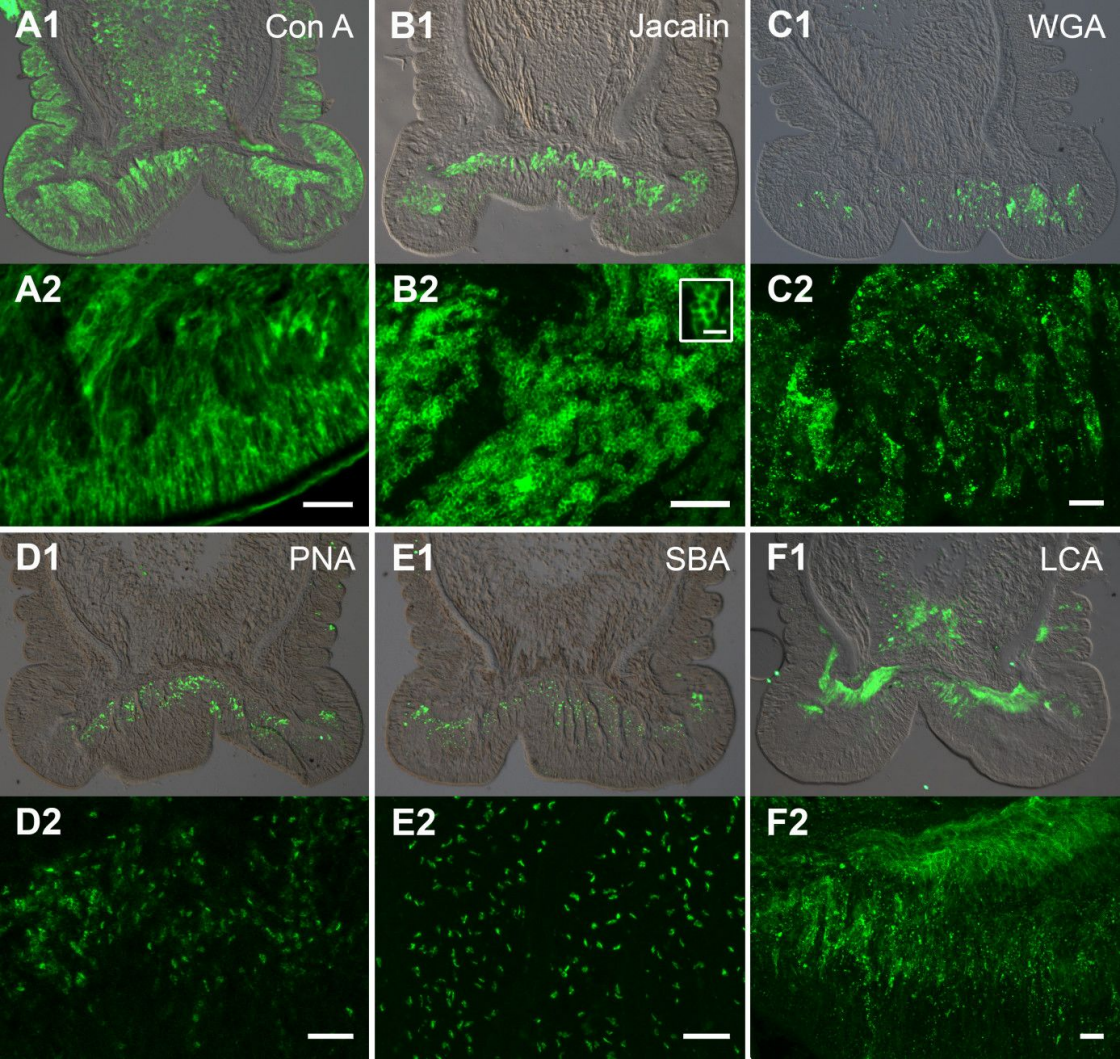
Lectin labelling of tube foot sections in *Asterina gibbosa* with (A1,A2) Con A, (B1,B2) Jacalin, (C1,C2) WGA, (D1,D2) PNA, (E1,E2) SBA, and (F1,F2) LCA. (A1–F1) Overlay of corresponding fluorescence- and differential interference contrast images. (A2–F2) Confocal *z*-projections of lectin labelling. Scale bars: 10 µm; (inset) 2 µm.

**Carbohydrate distribution in the disc epidermis and footprints.** To characterize carbohydrate moieties, we labelled tube foot sections from *A. gibbosa* with 24 specific lectins. Out of those, 15 have also been tested on *A. rubens* tube foot sections and footprint material [28]. Due to our interest in adhesion, we focussed on the description of labelling results within the disc epidermis. The summary of all results, indicating the intensity of staining on the different tissues is given in Table 1. Details on the sugar moieties recognized by the lectins are listed in (Supporting Information File 1, Table S1). Out of the 24 tested lectins, 15 led to a labelling in the disc epidermis (Figure 6, Supporting Information File 1, Figure S2,S3). Concanavalin A (Con A) strongly reacted with most tissues of the tube feet, except the connective tissue (Figure 6A1). In the disc epidermis, the area of the gland cells was strongly labelled from the gland cell bodies to the secretory pores (Figure 6A2). This staining throughout the gland cell necks indicated that the labelling corresponded to the secreted material. Only the lectin jacalin led to a similar overall staining of the gland cells (Figure 6B1). Higher resolution revealed that the jacalin labelling was restricted to ring-like structures (Figure 6B2). Due to their size and distribution, they most likely correspond to the outer rim of adhesive secretory granules (Figure 6B2 inset). However, whether they correspond to type 1 and/or 2 adhesive granules could not be distinguished. Similar to PNA, Jacalin binds to galactose (ß 1-3) *N*-acetylgalactosamine, but whereas PNA does not bind in the presence of sialic acid substitutions, Jacalin binds regardless of the presence of sialic acids (according to Manufacturer’s information, Vector laboratories). The Jacalin positive labelling therefore indicated galactose (ß 1-3) *N*-acetylgalactosamine with sialic acids in the outer parts of adhesive secretory granules.

**Table 1:** Overview of lectin labelling in *Asterina gibbosa* tube feet and footprints.

Lectin	Acronym	Preferred sugar specificity	Disc epidermis	Stem epidermis	Cuticle	CT	Myomesothel.	Footprints

Concanavaline A	Con A	αMan, αGlc	+++ (throughout glands)	+++	–	–	+++	+++
Jacalin	Jacalin	Galβ3GalNAc	+++ (outer rim vesicles)	–	–	–	++	–
Wheat germ agglutinin	WGA	GlcNAc	+++ (diffuse, basal area)	–	++	–	–	–
*Datura Stramonium* lectin	DSL	(GlcNAc)2-4	+++ (diffuse, basal area)	++	–	–	+	–
Peanut agglutinin	PNA	Galβ3GalNAc	+++ (elliptic dots)	++	–	–	+	–
Soybean agglutinin	SBA	α>βGalNAc	+++ (elliptic dots)	+	–	–	+	–
*Griffonia (Bandeiraea) simplicifolia* lectin I	GSL I	αGal, αGalNAc	+++ (elliptic dots)	+	–	–	–	–
*Vicia villosa* agglutinin	VVA	GalNAc	+++ (elliptic dots)	+++	–	–	–	–
Succinylated wheat germ agglutinin	sWGA	GlcNAc	+ (elliptic dots)	–	–	–	–	–
*Lens culinaris* agglutinin	LCA	αMan, αGlc	+++ (dots)	++	–	+++	++	–
*Pisum sativum* agglutinin	PSA	αMan, αGlc	+++ (dots)	–	–	+++	++	–
*Ricinus communis* agglutinin I	RCA I	Gal, GalNAc	++ (big, few)	–	–	–	–	–
*Griffonia (Bandeiraea) simplicifolia* lectin II	GSL II	α or βGlcNAc	++	++	–	–	+++	–
*Lycopersicon esculentum* (tomato) lectin	LEL	(GlcNAc)2-4	+	+	–	+	++	–
Elderberry bark Lectin	EBL	Neu5Acα6Gal/GalNAc	+	+	–	–	+	–
*Ulex europaeus* agglutinin 1	UEA 1	L-Fuc	–	+	–	–	–	–
*Maackia amurensis* lectin II	MAL II	Neu5Acα3Galβ4 GalNAc	–	–	–	++	+++	–
*Dolichos bilforus* agglutinin	DBA	αGalNAc	–	–	–	–	–	–
*Sambucus nigra* agglutinin	SNA	Neu5Acα6Gal/GalNAc	–	–	–	–	–	–
*Phaseolus vulgaris* erythro agglutinin	PHA-E	Galβ4GlcNAcβ2 Manα6(GlcNAcβ4) (GlcNAcβ4Manα3)Manβ4	–	–	–	–	–	–
*Phaseolus vulgaris* leuco agglutinin	PHA-L	Galβ4GlcNAcβ6 (GlcNAcβ2Manα3)Manα3	–	–	–	–	–	–
*Sophora Japonica* agglutinin	SJA	βGalNAc	–	–	–	–	–	–
*Erythrina cristagalli* lectin	ECL	Galβ4GlcNAc	–	–	–	–	–	–
*Solanum tuberosum* (potatoe) lectin	STL	(GlcNAc)2-4	–	–	–	–	–	–

Negative control, no lectin			–	–	–	–	–	–

+ weak labelling ,++ intermediate labelling, +++ strong labelling, – no labelling

Wheat germ agglutinin (WGA) and *Datura Stramonium* lectin (DSL) resulted in a strong labelling in the basal area of the gland cells, but in a weaker staining in the distal gland cell necks (Figure 6C1 and Supporting Information File 1, Figure 2A1–3). Neither labelling method allowed specific structures to be distinguished, but appeared diffuse and unevenly distributed, as shown for WGA (Figure 6C2). The five lectins, peanut agglutinin (PNA), Soybean agglutinin (SBA*), Griffonia (Bandeiraea) simplicifolia* lectin I , (GSL I), *Vicia villosa* agglutinin (VVA), and succinylated wheat germ agglutinin (sWGA), labelled the gland cells at the same area, but in contrast to WGA and DSL, led to a distinct staining of spherical to ellipsoid structures (Figure 6D,E, Supporting Information File 1, Figure 2B–D). For all five lectins, the structures were only observed in the basal area of gland cells and absent in the apical parts and the connective tissue (Figure 6D,E, Supporting Information File 1, Figure 2B–D). PNA led to a slightly more diffuse labelling (Figure 6D2) than the four other lectins. SBA, GSL I, and VVA labelled small ellipsoid structures, as shown for SBA (Figure 6E2). The labelling with sWGA resembled those with SBA, GSL I, and VVA, but with a much lower intensity and with a lower number of structures visible (Supporting Information File 1, Figure 2D). *Lens culinaris* agglutinin (LCA) and *Pisum sativum* agglutinin (PSA) strongly reacted with nerve strands (Figure 6F1, Supporting Information File 1, Figure 2E) and roundish forms in the disc epidermis (Figure 6F2). *Ricinus communis* agglutinin (RCA) labelled large ellipsoid structures in the distal part of gland cells (Supporting Information File 1, Figure 2F). However, only very few of these structures were observed. With *Griffonia (Bandeiraea) simplicifolia* lectin II (GSL II) and *Lycopersicon esculentum* (tomato) lectin (LEL) the epidermis of the disc and the stem were equally stained with intermediate and low intensity (Supporting Information File 1, Figure 3A,B). While the labelling with GSL II was homogeneous (Supporting Information File 1, Figure 3A), LEL resulted in a dotted staining (Supporting Information File 1, Figure 3B). Elderberry bark Lectin (EBL) resulted in a very weak labelling of the basal part of the disc epidermis (Supporting Information File 1, Figure 3C). *Ulex europaeus* agglutinin 1 (UEA 1) and *Maackia amurensis* lectin II (Mal II) both did not react with the disc epidermis, but labelled the stem epidermis (Supporting Information File 1, Figure 3D), and the connective tissue and the myomesothelium (Supporting Information File 1, Figure 3E), respectively. Out of the 24 lectins, 7 did not lead to a specific labelling on tube foot sections (Table 1). These lectins resulted in a very faint overall staining, as observed for the negative control (Supporting Information File 1, Figure 3F) (skipping the lectin and using only the Streptavidin-Dylight488 conjugate).

**Figure 7:**
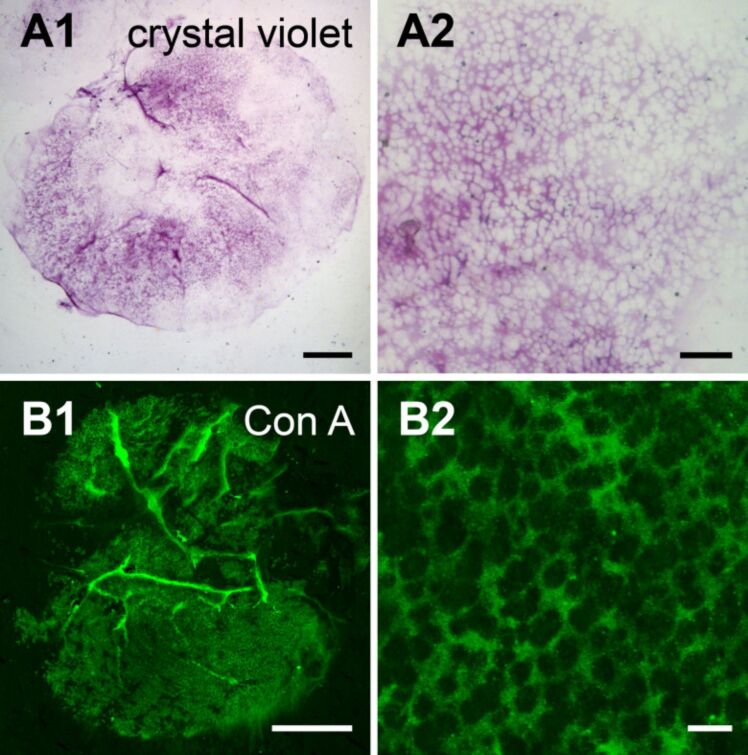
Structure of the footprints of *Asterina gibbosa* (light microscopy). (A1,A2) Cristal violet staining of a fresh footprint. (B1,B2) ConA labelling of a PFA fixed footprint, confocal *z*-projections. (B2) Confocal *z*-projection exclusively at the level of the meshwork, above the thin layer. Scale bars: (A1,B1) 100 µm; (A2) 20 µm; (B2) 5 µm.

Next, we tested if and which carbohydrates were present in the footprints. The footprints did not exhibit enough contrast to be directly investigated with light microscopy. Therefore, we used a crystal violet solution to check the presence and integrity of footprints on randomly selected microscope glass slides. The crystal violet staining corroborated the footprint structure described in previous sections (Figure 7A1). While the meshwork was always labelled with crystal violet, the thin film appeared not or weakly stained, varying between different areas of the footprints (Figure 7A2). From all tested 24 lectins, only ConA reacted strongly to fresh and paraformaldehyde (PFA)-fixed footprints (Figure 7B1). Both layers, the thin film and the meshwork, were equally strongly labelled. To visualize the meshwork, a confocal *z*-projection at the level of the meshwork was made (Figure 7B2). All other 23 lectins did not label fresh or PFA-fixed footprints. Whereas 15 lectins labelled structures in *A. gibbosa* tube feet sections, only Con A labelled the adhesive footprints. This discrepancy implied that most stained structures in the tube feet were not secreted. Indeed, only Con A and Jacalin labelled the whole area of the secretory gland cells, where the granules were prevalent. Although, Jacalin was found to label adhesive granules, it did not react with secreted footprints. It is possible that upon secretion conformational changes prevent the binding of the lectin. Other possible explanations are that although Jacalin reacts to the adhesive granules, the corresponding carbohydrates are not incorporated in the footprints or they may have been initially part of the footprints, but were lost during the detachment process.

Overall 11 lectins label the disc epidermis in *A. rubens* [28] and out of these five (Con A, WGA, SBA, GSL I, and RCA) also labelled the disc epidermis in *A. gibbosa*. However, in *A. gibbosa*, Con A was the only lectin that labelled footprints*,* whereas in *A. rubens* additionally WGA, RCA and DBA lead to a labelling there [28]. In both species, Con A strongly reacted with most tissues of the tube feet as well as with footprints. These results indicate the presence of glycoconjugates with α-linked mannose residues in the tissues and within the secreted adhesive material in both species.

Sugar moieties are present in the adhesive glands and/or adhesive material of various temporary attaching animals, like flatworms [44–45], sea urchins [27], *Hydra* [46], and cephalopods [47–48]. Similar to the two sea star species described in the previous section, Con A labels all tissues, including the adhesive gland cells, in the flatworm *M. lignano* [44]. Additionally, the outer rim of *M. lignano* adhesive vesicles was found to react with the lectin PNA, indicating the presence of galactose(ß 1-3) *N*-acetylgalactosamine [44]. In contrast to *A. gibbosa*, galactose(ß 1-3) *N*-acetylgalactosamine residues were also detected in *M. lignano* footprints (Lengerer pers. observation).

Many known proteins involved in temporary adhesion contain sugar-binding sites, like lectin-binding domains [8–10,49]. Although both carbohydrates and sugar binding sites are commonly found in temporary adhesives of marine and freshwater species, the specific role of glycosylation and carbohydrate-residues in the adhesion process is currently unknown. The characterization of sugar moieties in more species, like in the current study in *A. gibbosa,* might help to decipher the underlying mechanisms.

## Conclusion

The tube foot adhesive gland cells in *A. gibbosa* adult individuals were found to differ from the ones formerly described in attachment areas of developing individuals, highlighting that different adhesives are produced during developmental stages. The morphology of tube feet as well as the topography of secreted footprints share many similarities between the valvatid *A. gibbosa* and the formerly described forcipulatid *A. rubens.* In both species the adhesive material is produced by two types of adhesive gland cells and footprints consist of a thin layer with a meshwork on top of it. Additionally, in both species α-linked mannose residues were identified as part of the footprints. These resemblances might hint to a similar composition of the adhesive, likely caused by the adaptation to similar habitats. Despite these prevalent similarities, divergences were also identified. The type 2 adhesive granules in *A. gibbosa* are of same electron-density as type 1 granules and appear much more electron-dense than in *A. rubens*. Furthermore, only one lectin labelled footprints of *A. gibbosa,* suggesting a lower complexity in sugar moieties*.* Finally, antibodies raised against the adhesive protein Sfp1 from *A. rubens* did not cross-react with the adhesive gland cells or footprints in *A. gibbosa*. All these differences might be linked to the long evolutionary divergence between the two species. Further research on the adhesive composition of *A. gibbosa* will allow identification of conserved proteins and protein domains required for efficient attachment on rocky surfaces. In the long term, the characterization of its adhesive might help in designing new biomimetic glues.

## Experimental

### Maintenance of animals

Individuals of *Asterina gibbosa* (Pennant, 1777) were obtained from the Biological Sample Collection Service of the Station Biologique de Roscoff, France. They were kept in a marine aquarium with closed circulation (13 °C, 33‰ salinity).

### Footprint collection

Footprints for all experiments were collected on clean microscope glass slides. An adult animal was placed on a glass slide and then the slide was vigorously agitated under seawater for approximately 1–2 minutes, causing the animal to firmly attach. After voluntary detachment, the footprints were rinsed with MilliQ water to prevent the formation of salt crystals. Some of them were stained with a 0.05% (w/v) crystal violet solution in deionised water.

### Transmission electron microscopy (TEM)

For TEM, whole tube feet were fixed by immersion in 3% glutaraldehyde in cacodylate buffer (0.1 M. pH 7.8, with 1.55% NaCl) for 3 h at 4 °C. The tube feet were rinsed in cacodylate buffer (0.2 M. pH 7.8, with 1.84% NaCl) and then post-fixed in 1% osmium tetroxide in cacodylate buffer (0.1 M. pH 7.8, with 2.3% NaCl). After rinsing in cacodylate buffer, the tube feet were dehydrated in graded ethanol and embedded in Spurr resin. Semi-thin sections (1 µm) were performed with a Reichert Om U2 ultramicrotome equipped with a glass knife. The tube feet were sectioned either longitudinally or transversally. The sections were then stained with a 1:1 mixture of 1% aqueous solution of methylene blue in 1% sodium tetraborate and 1% aqueous solution of azur II. Ultrathin sections (80 nm) were cut with a Leica Ultracut UCT ultramicrotome equipped with a diamond knife. They were contrasted with uranyl acetate and lead citrate and observed with a Zeiss LEO 906E transmission electron microscope.

### Scanning electron microscopy (SEM)

Footprints were collected on clean glass coverslips and tube feet were cut off while being unattached. All samples were chemically fixed at room temperature in Bouin’s fluid for at least 24 h. All samples were then dehydrated in graded ethanol and dried by the critical-point method. They were mounted on aluminium stubs, coated with gold in a sputter-coater and observed with a JEOL JSM-7200F field emission scanning electron microscope.

### 3D confocal interference microscopy

Footprints were collected on clean microscope glass slides, rinsed with MilliQ water and air dried. Images were taken with a 3D confocal interference microscope (Sensofar PLu Neox, Sensofar Tech, Nederlands) equipped with a 5× (NA 0.15) objective. Confocal mode with 460 nm light was used to provide a high resolution 3D profile of the surface, with an image size of 256 × 256 data pixels points and a field of view of 847 × 847 µm^2^. The acquired images have been plane corrected. In addition, a soft prism filter correction was applied for image enhancement to recover parts that cannot be measured due to shadowing effects. An analytical software (SPIP 5.1.1, 2010, Image Metrology A/S) was used to determine the roughness parameters from the confocal images.

### Atomic force microscopy (AFM)

Footprints were collected on clean microscope glass slides, rinsed with MilliQ water and air dried. The footprints were then imaged in air and under ambient conditions with a Dimension Icon (Bruker Nano Inc., Santa Barbara, CA) using AFM in tapping mode. Tapping mode AFM was performed in amplitude modulation mode. The height of the cantilever position is constantly adjusted (via a feedback loop) to keep constant the ratio of the tip vibrational amplitude in contact with the sample surface to its oscillation free amplitude in air. This mode is well adapted for soft materials such polymers or biological samples. Due to the fact that forces existing between the surface and the tip are intermittent and small, the technique preserves the samples from damaging while scanning their surface. Silicon tips (NCH, Bruker Nano Inc) were calibrated on a stiff surface prior to experiments in order to quantify the tip–sample forces. The resonance frequency is about 320 kHz and their spring constant (determined by thermal tuning) is about 40 N/m. All the images were recorded with a resolution of 512 pixels/line using the Nanoscope software (Version 9.4). The scan rate was kept at 0.5 Hz. The Nanoscope Analysis image processing software (Version 1.90) was used for image analysis. The images were not filtered and only a 2nd order flattening procedure was applied to the raw data.

### Immunohistochemistry of tube foot sections and footprints

Antibody labelling was performed as previously described [8]. Briefly, tube feet were fixed in 4% PFA in phosphate buffered saline (PBS) for 24 h. They were subsequently dehydrated in graded ethanol, embedded in paraffin wax and cut longitudinally. After dewaxing and rehydration, antigen retrieval with a solution of 0.05% trypsin and 0.1% CaCl_2_ was performed for 15 min on 37 °C. Footprints were collected on microscope glass slides and fixed in 4% PFA in PBS overnight at room temperature. All samples were blocked in PBS containing 3% (w/v) bovine serum albumin (BSA) for 30 min at room temperature. Antibodies directed against Sfp1α and Sfp1β were diluted 1:100 in blocking solution and added to samples for 2 h at room temperature. Alexa Fluor 488-conjugated goat anti-rabbit immunoglobulins (Invitrogen) were applied 1:100 diluted in blocking solution for 1 h at room temperature. In the negative controls, no primary antibody was added and only the secondary antibody was applied. Samples were analysed with a Zeiss Axioscope A1 microscope.

### Lectin histochemistry of tube foot sections and footprints

Tube feet were fixed in Bouin’s fluid for 24 h. They were subsequently dehydrated in graded ethanol, embedded in paraffin wax, and cut longitudinally into 5 µm-thick sections with a Microm HM 240 E microtome or a Reichert Autocut 2030 microtome. The sections were dewaxed with two successive treatments with xylene for 10 min each. Afterwards the sections were rehydrated with graded ethanol. Footprints were collected on clean microscope glass slides and either used directly for labelling (fresh) or fixed in 4% PFA in PBS for 24 h. All samples were washed three times in Tris-buffered saline (TBS, pH 8.0) supplemented with 5 mM CaCl_2_ and 0.1% Triton (TBS-T). Unspecific background staining was blocked by pre-incubation in TBS-T containing 3% (w/v) BSA (BSA-T) for 2 h at room temperature. Biotinylated lectins were diluted in BSA-T to a final concentration of 25 µg/mL and applied to the sections for 2 h at room temperature. After three washes of 5 min each in TBS-T, the sections were incubated for 1 h in Dylight488-conjugated-streptavidin (Vector Laboratories) diluted 1:300 in BSA-T at room temperature. After three washing steps in TBS-T, the sections were mounted in Mowiol and analysed with a Leica DM5000 or a Zeiss Axioscope A1 microscope, or with a Leica SP5 II confocal scanning microscope. As the intensity of the labelling varied among different lectins (see Table 1), the images of the most strongly stained specimens (+++) and of weakly stained (+) specimens had to be taken at different exposure times to sufficiently visualize them without over- or underexposure. The negative control image was taken with the same, longer exposure time as for the weakly stained specimen.

## Supporting Information

File 1 Table S1: Overview of lectin binding specificity according to manufacturer Vector laboratories. Figure S1: Antibody labelling of tube foot sections from *Asterias rubens* (A,C) and *Asterina gibbosa* (B,D). Antibody directed against Sfp1α (A,B) and Sfp1β (C,D). Scale bars: 20 µm. Figure S2: Lectin labelling of tube foot sections from *Asterina gibbosa* with (A1-3) DSL, (B1-3) GSL I, (C1-3) VVA, (D1-3) sWGA, (E1-3) PSA, and (F1-3) RCA. Figure S3: Lectin labelling of tube foot sections from *Asterina gibbosa* with (A1-3) GSL II, (B1-3) LEL, (C1-3) EBL, (D1-3) UEA 1, and (E1-3) Mal II.
