# Supplementary material for: The structural and chemical basis of temporary adhesion in the sea star Asterina gibbosa
Source: Beilstein J Nanotechnol. 2018 Jul 30;9:2071–86. doi: 10.3762/bjnano.9.196 (PMC6122182; doi:10.3762/bjnano.9.196)
Supplement: File 1 — Table S1: Overview of lectin binding specificity according to manufacturer Vector laboratories. Figure S1: Antibody labelling of tube foot sections from Asterias rubens (A,C) and Asterina gibbosa (B,D). Antibody directed against Sfp1α (A,B) and Sfp1β (C,D). Scale bars: 20 µm. Figure S2: Lectin labelling of tube foot sections from Asterina gibbosa with (A1-3) DSL, (B1-3) GSL I, (C1-3) VVA, (D1-3) sWGA, (E1-3) PSA, and (F1-3) RCA. Figure S3: Lectin labelling of tube foot sections from Asterina gibbosa with (A1-3) GSL II, (B1-3) LEL, (C1-3) EBL, (D1-3) UEA 1, and (E1-3) Mal II. [file Beilstein_J_Nanotechnol-09-2071-s001.pdf]

Supporting Information

for

The structural and chemical basis of temporary adhesion in the sea star *Asterina gibbosa*

Birgit Lengerer[*1], Marie Bonneel[1], Mathilde Lefevre[2], Elise Hennebert[2], Philippe Leclère[3], Emmanuel Gosselin[4], Peter Ladurner[5] and Patrick Flammang[*1]

Address: [1]Biology of Marine Organisms and Biomimetics Unit, Research Institute for Biosciences, University of Mons, 23 Place du Parc, 7000 Mons, Belgium, [2]Cell Biology Unit, Research Institute for Biosciences, University of Mons, 23 Place du Parc, 7000 Mons, Belgium, [3]Laboratory for Chemistry of Novel Materials, Center for Innovation and Research in Materials and Polymers (CIRMAP), University of Mons, 20 Place du Parc, 7000 Mons, Belgium, [4]Laboratory of Physics of Surfaces and Interfaces (LPSI), University of Mons, 23 Place du Parc, 7000 Mons, Belgium and [5]Institute of Zoology and Center of Molecular Bioscience Innsbruck, University of Innsbruck, Technikerstr. 25, A-6020 Innsbruck, Austria

E-mail: Birgit Lengerer[*] - birgit.lengerer@umons.ac.be, Marie Bonneel - marie.bonneel@umons.ac.be, Mathilde Lefevre - mathilde.lefevre@umons.ac.be, Elise Hennebert - elise.hennebert@umons.ac.be, Philippe Leclère - philippe.leclere@umons.ac.be, Emmanuel Gosselin - emmanuel.gosselin@umons.ac.be, Peter Ladurner - peter.ladurner@uibk.ac.at, Patrick Flammang[*] - patrick.flammang@umons.ac.be
* Corresponding author

Antibody labelling of tube foot sections

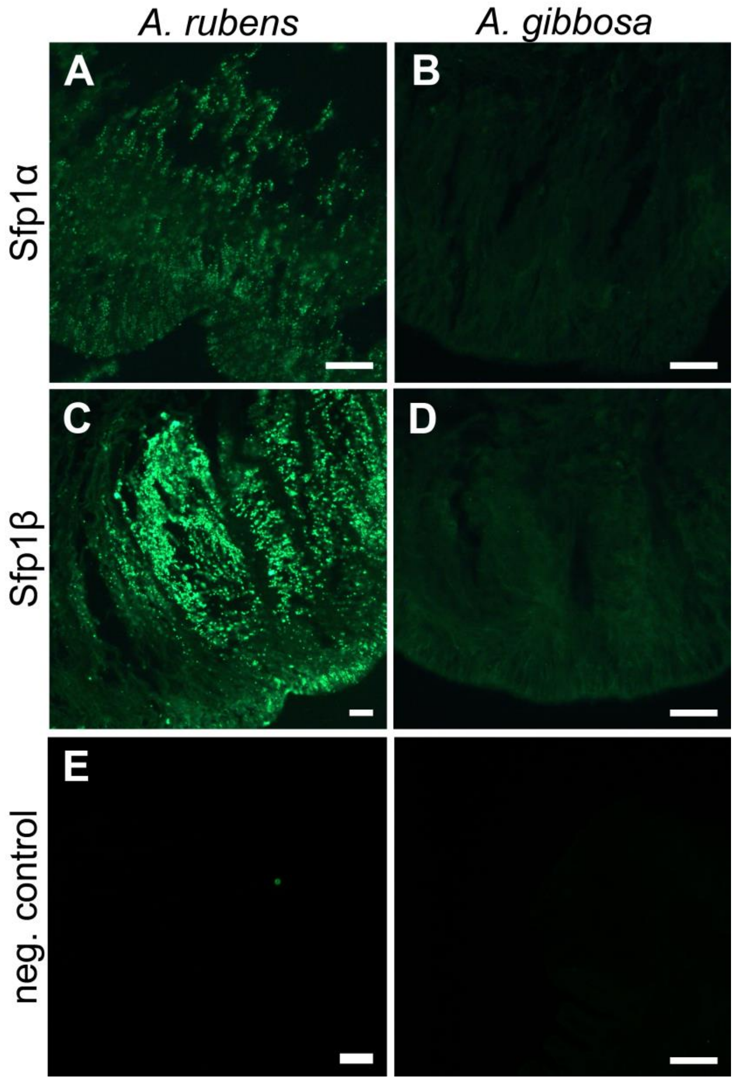

Figure S1: Antibody labelling of tube foot sections from *Asterias rubens and Asterina gibbosa*. Tube foot sections of *Asterias rubens* are on the left (A,C,E) and *Asterina gibbosa* to the right (B,D,F). Antibody directed against Sfp1α (A,B), against Sfp1β (C,D) and negative controls skipping the primary antibody (E,F). Scale bars: 20 µm.

Table S1: Overview of lectin binding specificity according to manufacturer Vector laboratories.

Lectin	Acronym	Preferred sugar specificity	Comments
Concanavaline A	Con A	αMan, αGlc	Recognizes α-linked mannose present as part of a core oligosaccharide in many serum and membrane glycoproteins
Jacalin	Jacalin	Galβ3GalNAc	Binds also in the presence of conjugated sialic acid.
Wheat germ agglutinin	WGA	GlcNAc	Preferable binds to dimer and trimers of GlcNAc. Can also bind terminal GlcNAc and chitobiose.
Datura Stramonium lectin	DSL	(GlcNAc)2-4	Preferable binds to chitobiose or chitotriose over single GlcNAc residues.
Peanut agglutinin	PNA	Galβ3GalNAc	Does not bind in the precence of conjugated sialic acid.
Soybean agglutinin	SBA	α>βGalNAc	Binds to oligosaccharide structures with terminal α- or β-linked *N*-acetylgalactosamine, and to a lesser extent, galactose residues.
Griffonia (Bandeiraea) simplicifolia lectin I	GSL I	αGal, αGalNAc	GSL I is a mixture of the five isolectins recognizing αGal and αGalNAc.
Vicia villosa agglutinin	VVA	GalNAc	Recognizes preferentially α- or β-linked terminal GalNAc, especially a single GalNAc residue linked to serine or threonine in a polypeptide.
Succinylated wheat germ agglutinin	sWGA	GlcNAc	This derivative does not bind to sialic acid residues, unlike the native form.
Lens culinaris agglutinin	LCA	αMan, αGlc	Recognizes sequences containing αMan residues but recognizes additional sugars as part of the receptor structure, giving it a narrower specificity than Con A.
Pisum sativum agglutinin	PSA	αMan, αGlc	Is nearly identical in structure and carbohydrate specificity to LCA.
Ricinus communis agglutinin	RCA I	Gal, GalNAc	Binds to Gal or GalNAc residues of membrane glycoconjugates.
Griffonia (Bandeiraea) simplicifolia lectin II	GSL II	α or βGlcNAc	Is unique in its ability to recognize exclusively α- or β-linked GlcNAc residues on the nonreducing terminal of oligosaccharides.
Lycopersicon esculentum (tomato) lectin	LEL	(GlcNAc)2-4	
Elderberry bark Lectin	EBL	Neu5Acα6Gal/GalNAc	Binds preferentially to sialic acid attached to terminal Gal in α-2,6 and to a lesser degree, α-2,3 linkage. This lectin does not appear to bind sialic acid linked to GalNAc.
Ulex europaeus agglutinin 1	UEA 1	L-Fuc	Binds to many glycoproteins and glycolipids containing α-linked fucose residues.
Maackia amurensis lectin II	MAL II	Neu5Acα3Galβ4 GalNAc	Binds only particular carbohydrate structures that contain sialic acid. Unlike SNA which seems to prefer structures with (α-2,6) linked sialic acid, MAL II appears to bind sialic acid in an (α-2,3) linkage.
Dolichos bilforus agglutinin	DBA	αGalNAc	
Sambucus nigra	SNA	Neu5Acα6Gal/Ga	Binds preferentially to sialic acid attached to terminal

agglutinin		lNAc	Gal in α-2,6 and to a lesser degree, α-2,3 linkage. This lectin does not appear to bind sialic acid linked to GalNAc.
Phaseolus vulgaris erythro agglutinin	PHA-E	Galβ4GlcNAcβ2 Manα6(GlcNAcβ 4)(GlcNAcβ4Man α3)Manβ4	
Phaseolus vulgaris leuco agglutinin	PHA-L	Galβ4GlcNAcβ6(GlcNAcβ2Manα3)Manα3	
Sophora Japonica agglutinin	SJA	βGalNAc	
Erythrina cristagalli lectin	ECL	Galβ4GlcNAc	Sialic acid substitution appears to prevent the lectin from binding.
Solanum tuberosum (potat oe) lectin	STL	(GlcNAc)2-4	Binds oligomers of GlcNAc and some bacterial cell wall oligosaccharides containing GlcNAc and *N*-acetylmuramic acid.

Sugar Abbreviations:

Fuc	L-Fucose
Gal	D-Galactose
GalNAc	*N*-Acetylgalactosamine
Glc	D-Glucose
GlcNAc	*N*-Acetylglucosamine
Man	Mannose
Neu5Ac	*N*-Acetylneuraminic acid (sialic acid)

Lectin labelling of tube foot sections from *Asterina gibbosa*.

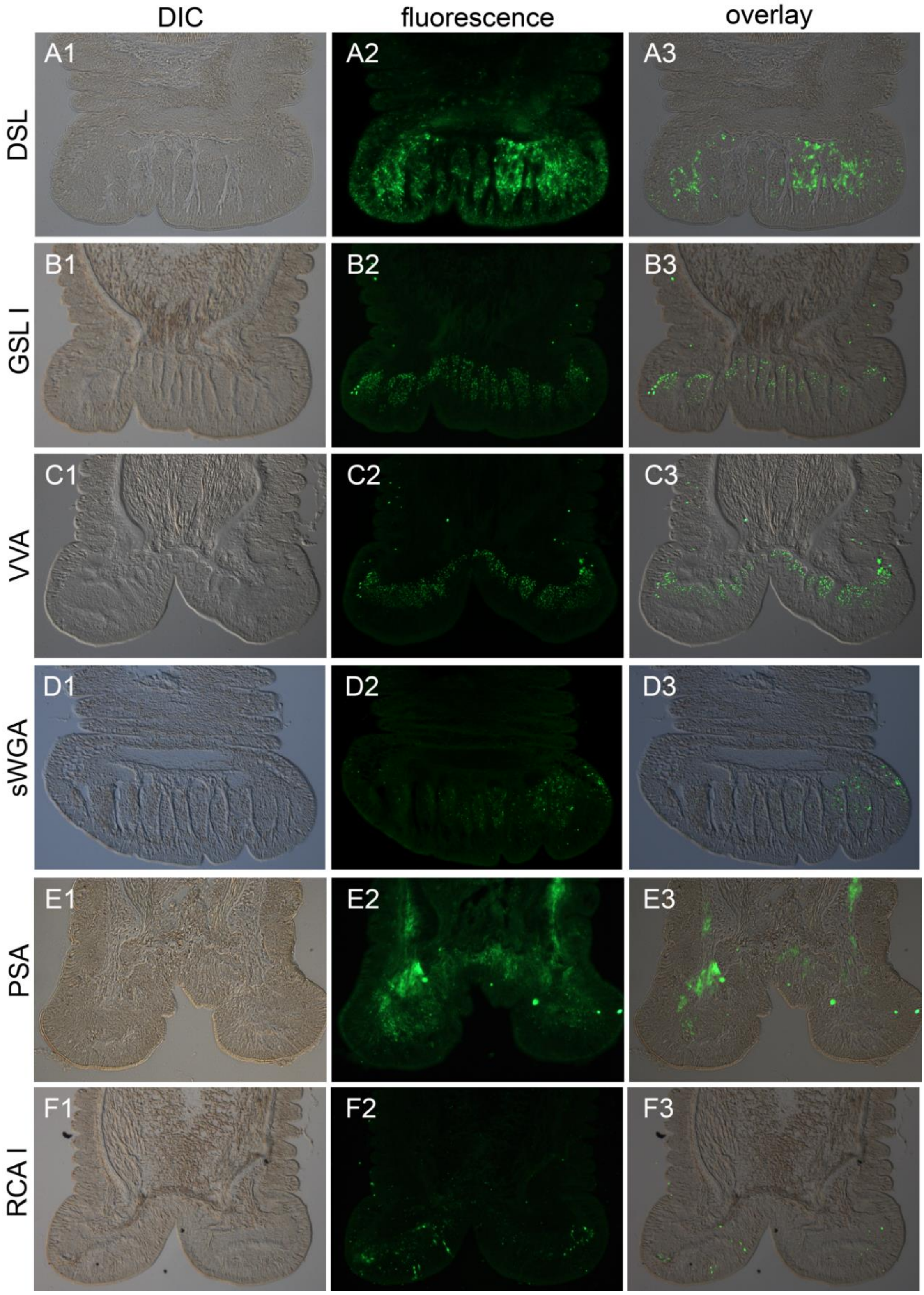

Figure S2: Lectin labelling of tube foot sections from *Asterina gibbosa;* with (A1-3) DSL, (B1-3) GSL I, (C1-3) VVA, (D1-3) sWGA, (E1-3) PSA, and (F1-3) RCA.

Lectin labelling of tube foot sections from *Asterina gibbosa*.

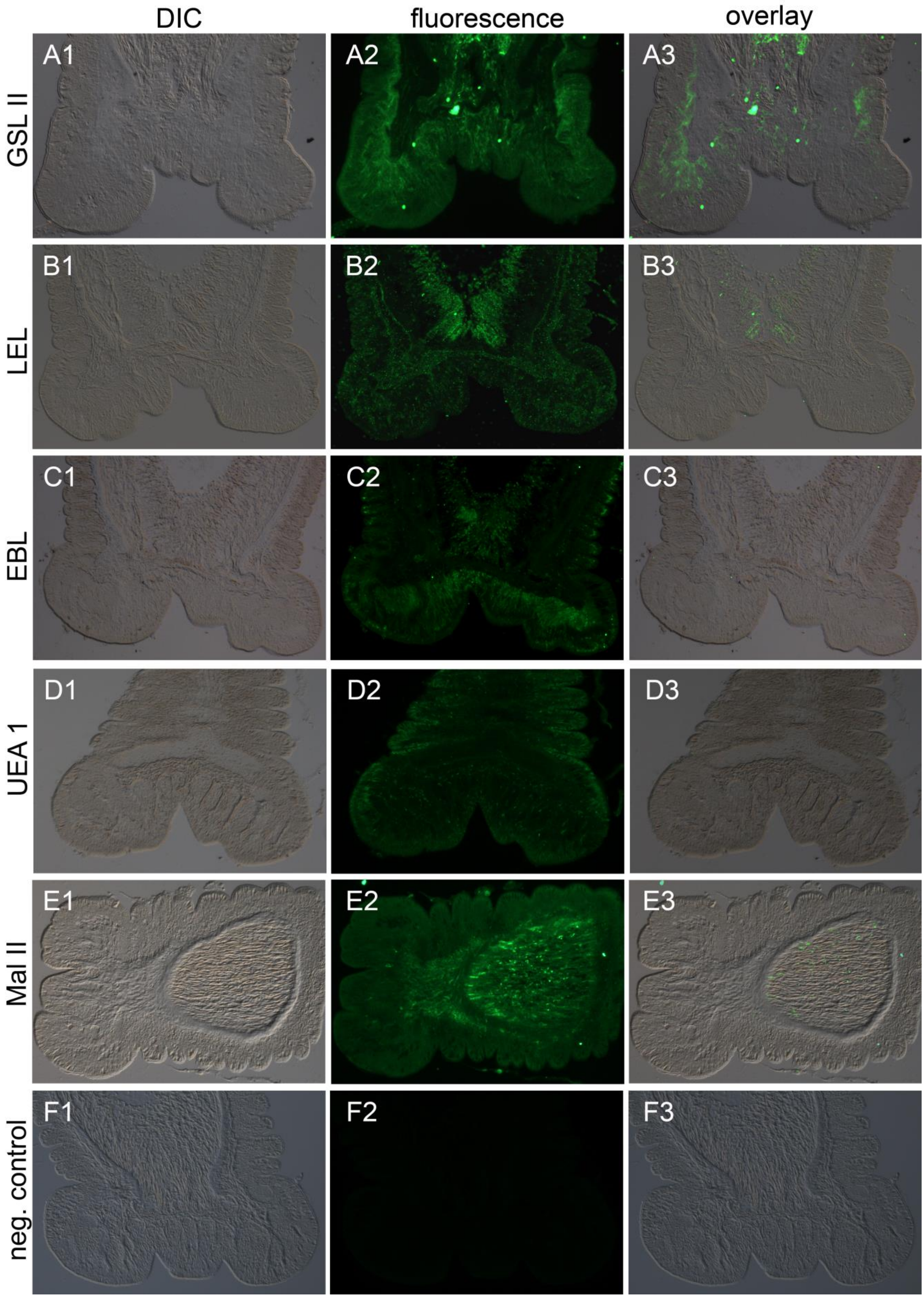

Figure S3: Lectin labelling of tube foot sections from *Asterina gibbosa*, with (A1-3) GSL II, (B1-3) LEL, (C1-3) EBL, (D1-3) UEA 1, (E1-3) Mal II and (F1-F3) negative control.
